# Generation of gene-modified goats targeting *MSTN* and *FGF5* via zygote injection of CRISPR/Cas9 system

**DOI:** 10.1038/srep13878

**Published:** 2015-09-10

**Authors:** Xiaolong Wang, Honghao Yu, Anmin Lei, Jiankui Zhou, Wenxian Zeng, Haijing Zhu, Zhiming Dong, Yiyuan Niu, Bingbo Shi, Bei Cai, Jinwang Liu, Shuai Huang, Hailong Yan, Xiaoe Zhao, Guangxian Zhou, Xiaoling He, Xiaoxu Chen, Yuxin Yang, Yu Jiang, Lei Shi, Xiue Tian, Yongjun Wang, Baohua Ma, Xingxu Huang, Lei Qu, Yulin Chen

**Affiliations:** 1 College of Animal Science and Technology, Northwest A&F University, Yangling 712100, China; 2 Shaanxi Provincial Engineering and Technology Research Center of Cashmere Goats, Yulin University, Yulin 719000, China; 3 Life Science Research Center, Yulin University, Yulin 719000, China; 4 College of Veterinary Medicine, Northwest A&F University, Yangling 712100, China; 5 MOE Key Laboratory of Model Animal for Disease Study, Model Animal Research Center of Nanjing University, National Resource Center for Mutant Mice, Nanjing 210061, China; 6 School of Life Science and Technology, ShanghaiTech University, Shanghai 201210, China

## Abstract

Recent advances in the study of the CRISPR/Cas9 system have provided a precise and versatile approach for genome editing in various species. However, the applicability and efficiency of this method in large animal models, such as the goat, have not been extensively studied. Here, by co-injection of one-cell stage embryos with Cas9 mRNA and sgRNAs targeting two functional genes (*MSTN* and *FGF5*), we successfully produced gene-modified goats with either one or both genes disrupted. The targeting efficiency of *MSTN* and *FGF5* in cultured primary fibroblasts was as high as 60%, while the efficiency of disrupting *MSTN* and *FGF5* in 98 tested animals was 15% and 21% respectively, and 10% for double gene modifications. The on- and off-target mutations of the target genes in fibroblasts, as well as in somatic tissues and testis of founder and dead animals, were carefully analyzed. The results showed that simultaneous editing of several sites was achieved in large animals, demonstrating that the CRISPR/Cas9 system has the potential to become a robust and efficient gene engineering tool in farm animals, and therefore will be critically important and applicable for breeding.

Genome-editing technologies rely on the use of engineered nucleases to induce cellular DNA repair mechanisms and introduce programmable, site-specific genetic modifications in diverse systems^1^. These programmable endonucleases include zinc finger nucleases (ZFNs), transcription activator-like effector nucleases (TALENs) and, most recently developed, the clustered regularly interspaced short palindromic repeats CRISPR-associated 9 (Cas9) system. The CRISPR/Cas9 system uses short, single-guide RNAs (sgRNA) that recognize the target DNA, then programmed the Cas9 towards targets that are complementary to the first 20 nucleotides of the sgRNA^2^. Compared with ZFNs and TALENs, the RNA-guided CRISPR/Cas9 system demonstrates its precious, versatile and robust merits for targeted genome editing in a variety of species, including model organisms, as well as crops and animals that are crucial to agriculture.

As one of the first domestic farm animals, the goat is one of the most important livestock species and provides a variety of products, including fiber, milk, meat, and hides. Furthermore, goats have also been used as a model in biomedical studies^3^,^4^,^5^. Although specific gene knockout strategies based on homologous recombination (HR) and somatic cell nuclear transfer (SCNT) have been established in goats^6^, precise gene modification of the goat genome is still challenging. In a previous study on disruption of four genes simultaneously in goat primary fibroblasts by the CRISPR/Cas9-mediated approach, only the myostatin (*MSTN*) knockout fibroblasts were achieved and resulted in live-born goats by SCNT^7^. However, an anti-biotic selection cassette is normally essential for isolating single-cell colonies from seeded donor cells, and reconstructed embryos have a low developmental potential, leading to a relatively low SCNT targeting efficiency (for example, 1–5% in pigs and >10% in cattle)^8^.

Co-injection of Cas9 mRNA and sgRNA into one-cell stage embryos has been demonstrated to be an efficient approach for the generation of genetically modified mice, rats, monkeys and pigs^9^,^10^,^11^,^12^, which encourages us to extend the application of this strategy to gene targeting in goats. In the present study, through co-injection of one-cell stage embryos of cashmere goats with sgRNAs of two functional genes (*MSTN* and *FGF5*) and Cas9 mRNA, targeted modifications of one or two genes were achieved at an efficiency around 26.5%. We also carefully analyzed the on- and off-target mutations of the targeted genes in the somatic tissues and gonads, providing comprehensive evidence for the efficiency and reliability of injection of zygotes with Cas9 mRNA and sgRNAs for the generation of gene-modified farm animals.

## Results and Discussion

### Design of sgRNAs

**Figure 1:**
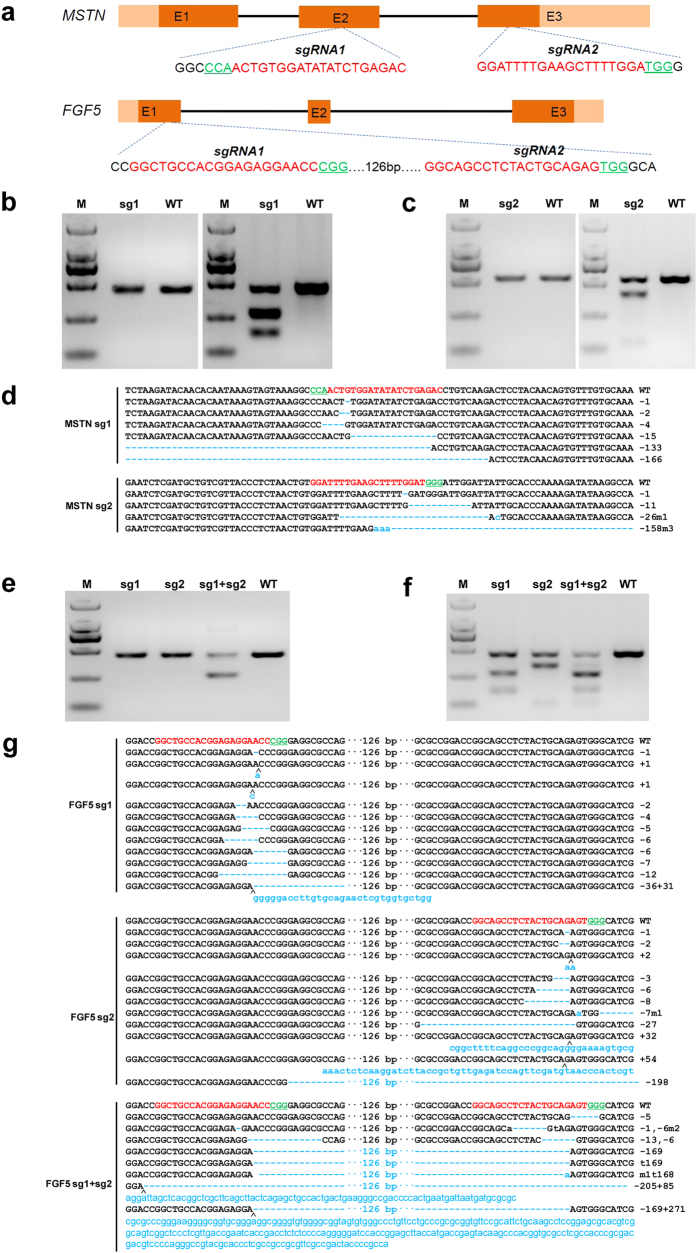
Evaluation of sgRNA:Cas9-mediated modifications of *MSTN* and *FGF5* in goat fibroblasts. (**a**) Schematic diagram of *MSTN* and *FGF5* partial protein coding region and the targeting loci of sgRNA:Cas9. sgRNAs targeting sites are presented in red. PAM sequences are highlighted in green and underlined. (**b**) Left panel, PCR products of the targeted exon 2 of *MSTN* from goat fibroblasts transfected with Cas9 and MSTN sgRNA1. Right panel, detection of sgRNA:Cas9-mediated on-target cleavage of *MSTN* by T7E1 cleavage assay. PCR products from left panel were subjected to T7E1 cleavage assay. M, marker; WT, wild type PCR product from fibroblasts that have not treated with CRISPR/Cas9. (**c**) Left panel, PCR products of the targeted exon 3 of *MSTN* from goat fibroblasts transfected with Cas9 and MSTN sgRNA2. Right panel, detection of sgRNA:Cas9-mediated on-target cleavage of *MSTN* by T7E1 cleavage assay. PCR products from left panel were subjected to T7E1 cleavage assay. (**d**) Sequences of modified MSTN alleles. Target sequences complementary to MSTN sgRNAs are in red text; the mutations are blue, lower case; insertions (+), deletions (−) or mutation (m) are shown to the right of each allele. (**e**) PCR products of the targeted exon 1 of *FGF5* from goat fibroblasts transfected with Cas9 and FGF5 sgRNA1, FGF5 sgRNA2 and FGF5 sgRNA1 & 2, respectively. (**f**) Detection of sgRNA:Cas9-mediated on-target cleavage of *FGF5* by T7E1 cleavage assay. PCR products from (e) were subjected to T7E1 cleavage assay. (**g**)Sequences of modified *FGF5* alleles. Target sequences complementary to *FGF5* sgRNAs are in red text; the mutations are blue, lower case; insertions (+), deletions (−), mutation (m) or turnover (t) i shown to the right of each allele.

The Shannbei cashmere goat is a cultivated dual-purpose breed that provides both meat and fibers (fine cashmere). In an attempt to improve the performance of cashmere goats considering both meat and cashmere production purposes, two genes that are associated with muscle development (*MSTN*) and hair length (*FGF5*), were selected as target genes. MSTN is a secreted growth differentiation factor that inhibits muscle differentiation and growth. Function loss of *MSTN* is known to cause an increased muscle mass phenotype in several mammals, including mice, dogs, cattle and humans^13^,^14^,^15^,^16^. Fibroblast growth factor 5 (FGF5), a secreted signaling protein during the hair growth cycle, inhibits hair growth by blocking dermal papilla cell activation^17^, and is regarded as the causative gene underlying the *angora* phenotype (long hair coat) in mice^18^. Mutations in *FGF5* underlie trichomegaly (excessively long eyelashes) in humans^19^, as well as being associated with hair length in other mammal species such as cats^20^, dogs^21^ and donkeys^22^. *MSTN* and *FGF5* were therefore selected to generate gene-modified cashmere goats with both genes disrupted by the CRISPR/Cas9 system. To ensure the successful targeting, two sgRNAs independently targeting exons 2 and 3 of *MSTN*, and two sgRNAs targeting the first exon of *FGF5*, were selected as described^23^ (Fig. 1a).

### Efficiency of the CRISPR/Cas9 System in Fibroblasts

Although microinjection of zygotes with Cas9 mRNA and sgRNA had not previously been tested in goats, the successful application of this system in embryos of other species^12^,^24^, suggested that this system could work in goat embryos. To verify this possibility, we overexpressed the Cas9 nuclease and sgRNAs targeting *MSTN* and *FGF5* in goat fibroblasts isolated from goat foetus. Subsequently, genomic DNA was extracted from fibroblasts 72 h after transfection, and screened for the presence of site-specific gene modification by PCR amplification of the region around the targeted site (Fig. 1b, c and e) using a T7 endonuclease I (T7E1) cleavage assay. The T7E1 cleavage bands were visible in the targeted genes (Fig. 1b, c and f). The cleavage was characterized further by Sanger sequencing, which displayed overlapped peaks in the sequencing chromatographs (Fig. 1d,g), suggesting different genotypes from target modifications. Distinguishable indels were observed at the target sites of *MSTN* and *FGF5* with a range of mutation sizes (Fig. 1d,g, Supplementary Table S1). These data demonstrated that the designed sgRNAs work efficiently with Cas9 on targeted genes in the cultured goat fibroblasts.

### Generation of Gene-Modified Goats

**Figure 2:**
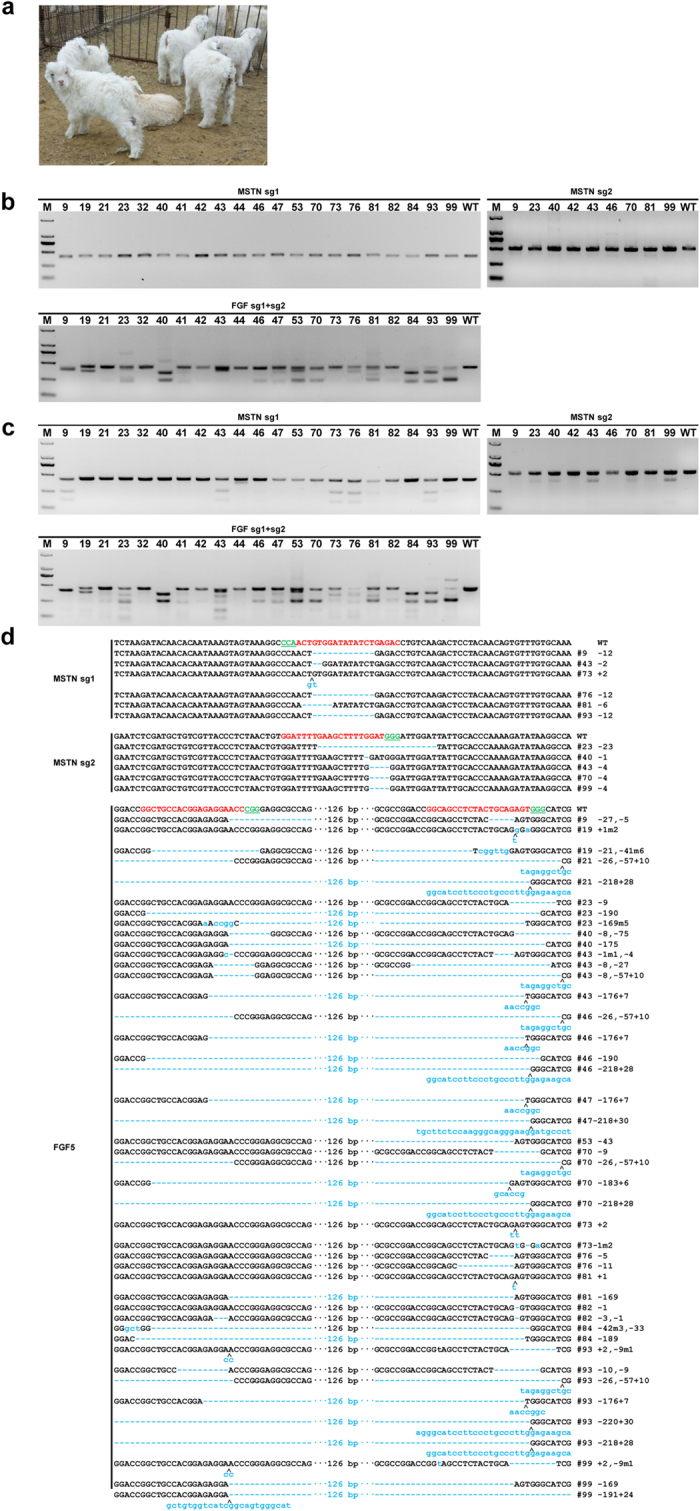
Detection of sgRNA:Cas9-mediated modifications of *MSTN* and *FGF5* in lambs and tissues. (**a**) Photographs of 30-day-old gene-modified lambs (Photo taken by X.W.). (**b**) PCR products of the targeted region of *MSTN* and *FGF5* from founder goats co-microinjected with a mixture of Cas9 mRNA and sgRNAs. (**c**) Detection of sgRNA:Cas9-mediated on-target cleavage of *MSTN* and *FGF5* by T7E1 cleavage assay. All PCR products from (**b**) were subjected to T7E1 cleavage assay. All the samples were digested by T7E1, suggesting that all founders carry *MSTN* and *FGF5* mutations. (**d**) Sequencing results of modified *MSTN* and *FGF5* loci detected in lambs.

To establish target modification in goats, a total of 926 early zygotes (one-cell stage) were surgically collected from 79 naturally mated ewes; ~11.72 zygotes were obtained from each donor. The Cas9 mRNA and sgRNA mixture targeting *MSTN* and *FGF5* was injected into the cytoplasm of the embryos at the one-cell stage. After the injected embryos developed to the two-cell stage (~24 h, 37 °C), only 416 (48.25%) of the 862 injected embryos were transferred into 137 pseudopregnant mothers. On average, 3.04 injected embryos were transferred to each recipient (Table 1). Of the 137 recipient ewes, 64 pregnancies (46.7%) were established according to observation of the estrus cycles. After full-term gestation, which lasted around 150 days, 93 lambs from surrogates were successfully delivered, 14 of which died immediately after birth (Fig. 2a and Table 1).

Genomic DNA was isolated from aborted, dead and live lambs. In total, 98 individuals (including 93 delivered and 5 aborted) were used for genotyping. The sgRNA:Cas9-mediated genome modifications were first screened using the genomic DNA as described above. Genotyping was performed by PCR amplification, T7E1 assay, and TA-cloning sequencing. The additional bands were observed by PCR amplification of the target region in some of the infant goats (Fig. 2b), indicating that genomic modification occurred in these goats. Then, the PCR products of all the animals were subjected to the T7E1 cleavage assay (Fig. 2c). Impressively, the cleavage products for *MSTN* were observed in 15 infants (15.3%) and *FGF5* in 21 infants (21.4%), with 10 lambs showing disruption of two genes simultaneously (Table 2). In total, 26 lambs (26/98, 26.5%) demonstrated the disruption of one or two genes, indicating efficient genomic modifications in the infant goats. As expected, different types of indels were observed, and further confirmed by Sanger sequencing (Fig. 2d).

Currently, there are two descriptions about generation of genetically modified goat and sheep via the CRISPR/Cas9 system. Ni *et al.* reported the application of the CRISPR/Cas9 system to disrupt four genes (*MSTN*, *NUP*, *PrP*, and *BLG*) in goat primary fibroblasts, and generated live-born goats via SCNT approach for the single *MSTN* gene. Only the *MSTN* biallelic mutations were used for SCNT; seven pregnancies were yielded from 21 transfers, and two live-born goats were eventually obtained^7^. Through co-injection of Cas9 mRNA and sgRNA targeting a single gene-*MSTN*, Han *et al.* described the successful generation of *MSTN* knockout sheep at a targeting efficiency of 5.7% (2/35)^25^. Taken together, our results support that direct microinjection of Cas9 mRNA and sgRNAs into one-cell stage embryos is efficient in goats, and could be applied to produce gene-modified animals.

### Cas9-Mediated Genome Targeting Extensively Integrates into Different Tissues of Goats

**Figure 3:**
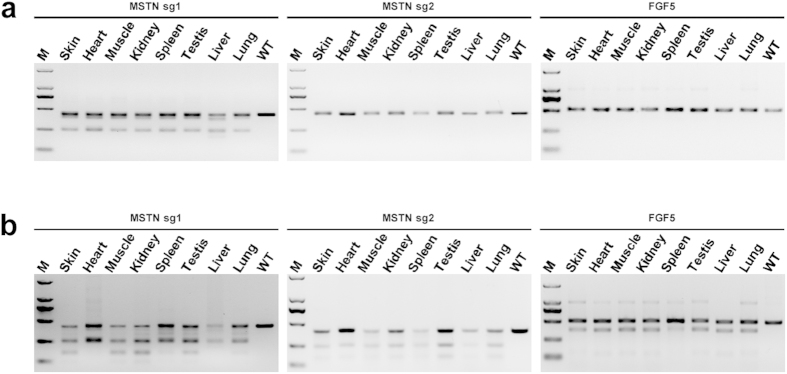
Detection of sgRNA:Cas9-mediated targeting in different tissues. (**a**) Dead goat #D24 was chose for tissue distribution analysis of on –target mutations. PCR products of the targeted regions of *MSTN* and *FGF5* from seven tissues. (**b**) Detection of sgRNA:Cas9-mediated on-target cleavage of *MSTN* and *FGF5* by T7E1 cleavage assay. All PCR products from (**a**) were subjected to T7E1 cleavage assay. All the samples were digested by T7E1, suggesting that all the 7 tested tissues carry *MSTN* and *FGF5* mutations.

To further evaluate the integration of the Cas9-mediated modifications into the derivatives of three germ layers, we performed extensive analysis of target mutagenesis in seven different somatic tissues (heart, liver, spleen, lung, kidney, skin, and muscle) and testis from the aborted and dead lambs. PCR amplification and T7E1 cleavage assay showed similar cleavage patterns among different tissue (Fig. 3a,b), suggesting that the Cas9-mediated mutations did occur in various goat tissues.

### Off-Target Detection

**Figure 4:**
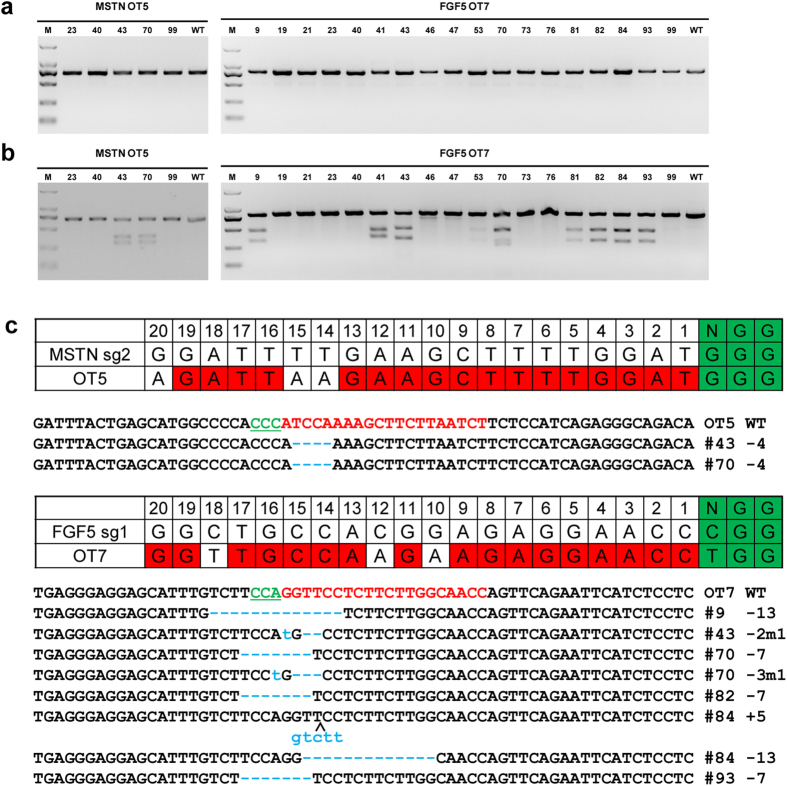
Detection of the *MSTN* and *FGF5* sgRNA:Cas9-mediated off-target cleavages *in vivo.* (**a**) PCR products of the potential off-target sites of *MSTN* and *FGF5* sgRNA:Cas9 from founder lambs. A total of 13 potential off-target sites most homologous to *MSTN* and *FGF5* sgRNA were named OT1 to OT13. OT5 and OT7 were selected and PCR amplified from genomic DNA from founders. (**b**) Detection of sgRNA:Cas9-mediated off-target cleavage of *MSTN* and *FGF5* by T7E1 cleavage assay. All PCR products from (**a**) were subjected to T7E1 cleavage assay. (**c**) Sequencing results of PCR products.

Previous studies have described that off-target mutagenesis occurred in Cas9 medicated human cell lines^25^, mice^26^ and zebrafish^27^, suggesting that mismatches between the sgRNA and target DNA exist due to Cas9-mediated DNA cleavage^25^. To verify whether off-target mutations occurred in these Cas9 mediated goats, we predicted putative off-target sites using SeqMap^28^ and assessed the off-target mutations in both fibroblasts and founder animals. A total of 13 (6 for *MSTN* and 7 for *FGF5*) most potential off-target sites were predicted across the goat genome (Supplementary Table S2). Out of the 13 off-target sites, two sites (OT5 and OT7) showed off-target modifications in goat fibroblast cells (Supplementary Table S3, Fig S1). The remaining 11 off-target sites were therefore excluded for further examination in the founder animals. Subsequently, the PCR products at OT5 and OT7 were subjected to the T7E1 cleavage assay using the DNA from founder animals, we found that mutations at these two sites indeed occurred in some of the founder animals (Fig. 4a,b). To further verify the off-target cleavage events, PCR products from *MSTN*-disrupted animals and *FGF5*-disrupted animals were subjected to Sanger sequencing, showing #43, #70 of *MSTN* mutated founders and #9, #43, #70, #82, #84, #93 of *FGF5* mutated founders had off-target modification (Fig. 4c). Except #41 of *FGF5*, which had a TGG-------GGA/AGGAGGGGTGGGG SNP 113 bp before OT7, no mutations were found in #53 and #81 of *FGF5* mutated founders, indicating the slight off-target efficiency. In addition, #46 and #47 of *FGF5* showed false positive cut bands due to confirmed poly-T structure. Considering the off-target effects are site-dependent, we believe the off-target mutagenesis can be avoided by choosing a single or several sites.

Taken together, we present here the successful generation of gene-modified goats targeting multiple genomic loci via the CRISPR/Cas9 approach. We further carefully analyzed the sgRNA:Cas9-mediated on- and off-target mutation in various somatic tissues, as well as testis, providing detailed information about generating gene-modified goats by injection of zygotes with Cas9 mRNA and sgRNAs.

## Materials and Methods

### Ethical statement

Eleven rams (2–3 years old, body weight: 30–50 kg) and 216 ewes (79 donors and 137 recipients, 2–3 years old, body weight: 24–40 kg) were used in this study. The animals were regularly maintained in the Shaanbei Cashmere Goat Farm of Yulin University. All the protocols involving the use of animals were in accordance with approved guidelines of the Animal Care and Use Committee of the Northwest A&F University (Approval ID: 2011ZX08008-002).

### Design of Cas9/sgRNA

To construct the recombinant vector for the preparation of sgRNA by *in vitro* transcription, the two complementary DNA oligos shown in Supplementary Table S4 were annealed to be double-stranded, and then sub-cloned into the pUC57-T7-gRNA vector as described^9^. The constructed recombinant vector was completely linearized by DraI endonuclease and used as the template. sgRNAs were produced by *in vitro* transcription using the MEGAshortscript kit (Ambion) and purified using the MEGAClear kit (Ambion) according to the manufacturer’s instructions. Using the Cas9 mRNA *in vitro* transcription vector (Addgene No. 44758) as templates, Cas9 mRNA was produced and purified according to description^9^. The sequence of both the Cas9 and sgRNAs targeting *MSTN* and *FGF5* are listed in Supplementary Table S5.

### Cas9/sgRNA efficiency test in goat fetal fibroblasts

We established a fibroblast cell line from 40-day-old goat fetus trunk tissue. The fibroblasts went through five passages cultured in DMEM medium (Gibco) supplemented with 10% FBS (Gibco) and 1% penicillin streptomycin (Gibco) to achieve 80–90% confluency on the day of transfection. The transfection procedure was carried out using Lipofectamine 2000 Reagent (Invitrogen) according to the manufacturer’s instructions. Briefly, goat fibroblasts were transfected with MSTN sgRNA1 (0.8 ug), MSTN sgRNA2 (0.8 ug), FGF5 sgRNA1 (0.8 ug), FGF5 sgRNA2 (0.8 ug) and FGF5 sgRNA1 & 2 (0.4 ug/each), along with 0.8 μg of Cas9 plasmid by Lipofectamine 2000 in a 24-well culture plate. 24 h after transfection, 10 mg /mL of blasticidine S hydrochloride was added to the medium at a 1:1000 dilution for 24 h. Genomic DNA was extracted from fibroblasts 72 h after transfection using a saturated solution of phenol and chloroform, then the DNA was precipitated with alcohol and sodium acetate. Subsequently, a T7E1 cleavage assay was performed as described by Shen *et al.*^9^. Briefly, the targeted fragments were amplified by PrimerSTAR HS DNA polymerase (TaKaRa, DR010A) from the genomic DNA, then purified with a PCR cleanup kit (Axygen, AP-PCR-50). The primers for amplifying *MSTN* and *FGF5* targeted fragments are listed in Supplementary Table S6. The purified PCR products were denatured and re-annealed in NEBuffer 2 (NEB) using a thermocycler (BioRad). The PCR products were digested with T7E1 (NEB, M0302L) for 30 min at 37 °C and then separated on a 2.5% agarose gel. The PCR products with mutations detected by the T7E1 cleavage assay were then sub-cloned into T vector (Takara). For each sample, the colonies were picked randomly and sequenced with the M13F (–47) primer: 5′-CGC CAG GGT TTT CCC AGT CAC GAC-3′).

### Production of gene-modified goats via zygote injection with Cas9/sgRNA

Healthy ewes with regular estrus cycles were selected as donors for zygote collection. Zygotes were collected through surgical oviduct flushing from the donors by estrus synchronization and superovulation treatment as we previously described^30^,^31^. In brief, donors were treated with EAZI-BREED CIDR Sheep and Goat Device (CIDR, contain progesterone 300 mg) by inserting in vagina for 14 days, and the superovulation was performed 60 hours prior to CIDR Device removal. A total of 260 mg follicle stimulating hormone (FSH) (Folltropin®-V) was administered by intramuscular injection in 7 dosages, at 12 h intervals (the first dose was 70 mg and other doses were decrease progressively to 25 mg). And subsequently injected with 0.1 mg cloprostenol after 60 hours from FSH was administered. Estrous detection was carried out 12 h after CIDR withdrawal and mating were repeated at 8 h intervals.

Goat zygotes at the one-cell stage (around 14 h post-fertilization) were surgically collected and were immediately transferred into TCM199 medium (Gibco, NY, USA). Cas9 mRNA (2  ng/μL) and sgRNAs (5 ng/μL for each sgRNA) targeting *MSTN* and *FGF5* were mixed and injected into the cytoplasm of fertilized oocytes using the FemtoJect system (Eppendorf, Hamburg, Germany). The injection pressure, injection time and compensatory pressure were 45 kpa, 0.1 s and 7 kpa, respectively. Microinjection was conducted in manipulation medium TCM199 on the heated platform of the Olympus micromanipulation system ON3. After injection, the zygotes were cultured in Quinn’s Advantage Cleavage Medium (Sage Biopharma, NJ, USA) for 24 h at 37 °C, and then transferred to Quinn’s Advantage Blastocyst Medium (Sage Biopharma, NJ, USA) at 37 °C, 5% concentration of carbon dioxide and saturated humidity conditions. The surrogate animals for transfer were determined according to their oestrus cycles. About three divisive embryos were transferred into the ampullary-isthmic junction of the oviduct of the surrogate ewes. Pregnancy was determined by observing the oestrus behaviors of surrogate ewes every ovulation circle.

### T7E1 cleavage assay and sequencing

Samples including blood, heart, liver, spleen, lung, kidney, skin, testis, and muscle, were collected and digested in lysis buffer (0.4 M NaCl, 2 μM EDTA, 1% SDS, 10 μM Tris-HCl, and 100 μg/ml Proteinase K). The genomic DNA of the sample was extracted from the lysate by the phenol-chloroform protocol, and recovered by alcohol precipitation. A T7E1 cleavage assay was performed as described^9^.

### Off-target assay

To determine the site-specific cleavage of the CRISPR-Cas9 system *in vivo*, the potential off-target loci were searched using an open tool, SeqMap^29^. The mismatch parameter for the target sequence was set as 5. ‘NGG’ and ‘NAG’ were chosen as the protospacer adjacent motifs (PAM). The sites with 7 bp conserved proximal to PAM and total mismatches <5, and sites with total mismatches <4, were chosen as potential off-target sites for subsequent testing. The selected potential off-target sites were initially PCR-amplified using genomic DNA from cultured goat fibroblasts. The PCR products were then subjected to a T7E1 cleavage assay. The potential off-target sites yielding the typical cleavage bands were considered as candidates and further evaluated in the founder animals by PCR amplification, T7E1 cleavage assay and TA sequencing. The information on the off-target loci and primer pairs used are listed in Supplementary Tables S7.

## Additional Information

**How to cite this article**: Wang, X. *et al.* Generation of gene-modified goats targeting *MSTN* and *FGF5* via zygote injection of CRISPR/Cas9 system. *Sci. Rep.* **5**, 13878; doi: 10.1038/srep13878 (2015).

## Supplementary Material

Supplementary Information

## Figures and Tables

**Table 1:** Summary of production of gene-modified goats via CRISPR-Cas9.

Goats forzygotecollection	Collectedembryos	Cas9-sgRNA injectedembryos (one-cell stage)		Recipientgoats^a^	Newborns	Alive goats	Gene-modified goats
		Injectedembryos	Transferredembryos				
79	926	862	416	137	93	79	26

^a^Five surrogate ewes had aborted.

**Table 2:** The efficacy of Cas9-mediated modifications in goat fibroblasts and tested individuals.

Target gene	Fibroblasts	Tested individuals
*MSTN*	15/26 (57.7%)	15/98 (15.3%)
*FGF5*	11/18 (61.1%)	21/98 (21.4%)
*FGF5*&*MSTN*	−	10/98 (10.2%)
