# Supplementary material for: Generation of gene-modified goats targeting MSTN and FGF5 via zygote injection of CRISPR/Cas9 system
Source: Sci Rep. 2015 Sep 10;5:13878. doi: 10.1038/srep13878 (PMC4564737; doi:10.1038/srep13878)
Supplement: Supplementary Information [file srep13878-s1.doc]

**Generation of gene-modified goats targeting *MSTN* and *FGF5* via zygote injection of CRISPR/Cas9 system**

Xiaolong Wang^1§^, Honghao Yu^2,3§^, Anmin Lei^4§^, Jiankui Zhou^5,6§^, Wenxian Zeng^1^, Haijing Zhu^2,3^, Zhiming Dong^6^, Yiyuan Niu^1^, Bingbo Shi^1^, Bei Cai^1^, Jinwang Liu^2,3^, Shuai Huang^2,3^, Hailong Yan^1,2,3^, Xiaoe Zhao^4^, Guangxian Zhou^1^, Xiaoling He^1^, Xiaoxu Chen^1^, Yuxin Yang^1^, Yu Jiang^1^, Lei Shi^2,3^, Xiue Tian^1^, Yongjun Wang^1^, Baohua Ma^4*^, Xingxu Huang^5,6*^, Lei Qu^2,3*^, Yulin Chen^1*^

^1^College of Animal Science and Technology, Northwest A&F University, Yangling 712100, China.

^2^Shaanxi Provincial Engineering and Technology Research Center of Cashmere Goats, Yulin University, Yulin 719000, China.

^3^Life Science Research Center, Yulin University, Yulin 719000, China.

^4^College of Veterinary Medicine, Northwest A&F University, Yangling 712100, China.

^5^MOE Key Laboratory of Model Animal for Disease Study, Model Animal Research Center of Nanjing University, National Resource Center for Mutant Mice, Nanjing 210061, China.

^6^School of Life Science and Technology, ShanghaiTech University, Shanghai 201210, China.

^§^These authors contributed equally to this work.

*Correspondence: [chenyulin@nwafu.edu.cn](mailto:chenyulin@nwafu.edu.cn) (Y.C), [ylqulei@126.com](mailto:ylqulei@126.com) (L.Q), [xingxuhuang@mail.nju.edu.cn](mailto:xingxuhuang@mail.nju.edu.cn) (X.H), [mabh@nwafu.edu.cn](mailto:mabh@nwafu.edu.cn) (B.M)


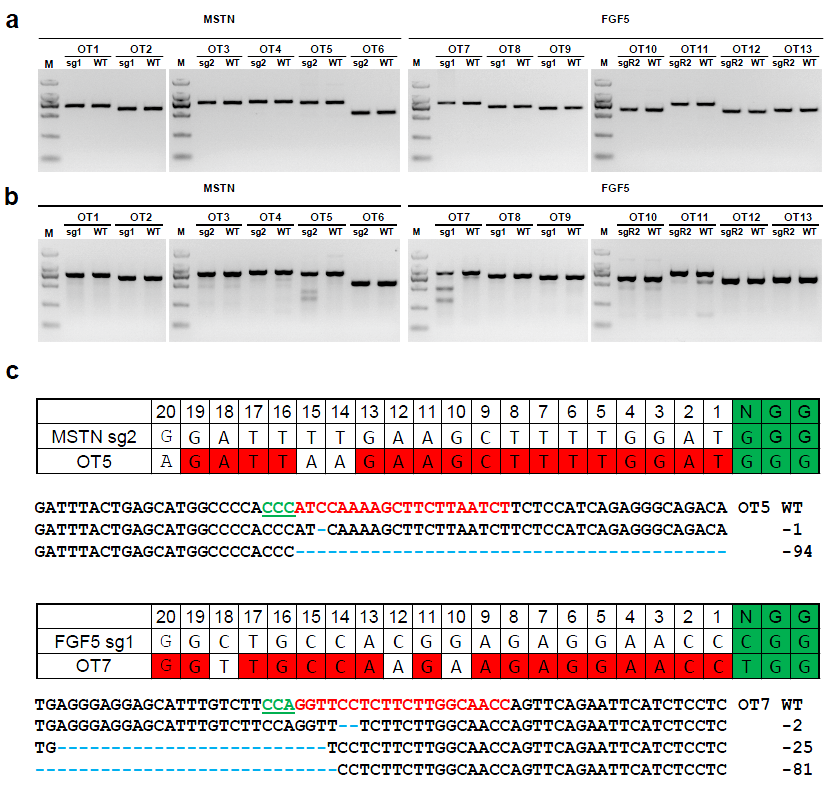
**Supplementary Fig. S1** **Off-target analysis on goat fibroblasts.**

(a) PCR products of the potential off-target sites of *MSTN* and *FGF5* sgRNA. A total of 13 potential off-target sites most homologous to *MSTN* and *FGF5* sgRNA were named OT1 to OT13.

(b) Detection of sgRNA:Cas9-mediated off-target cleavage of *MSTN* and *FGF5* by T7E1 cleavage assay. All PCR products from (a) were subjected to T7E1 cleavage assay.

(c) Sequencing results of off-target cleavage.

**Supplementary Table S1** Sequencing and T7E1 analysis summary of on-target on fibroblasts.

| No. | PCR product sequencing | T7E1 | Sequenced Colonies | Mutations |
| --- | --- | --- | --- | --- |
| MSTN-sgRNA-1 on fibroblasts | - | positive | 2 | -1 |
|  |  |  | 1 | -2 |
|  |  |  | 1 | -4 |
|  |  |  | 1 | -15 |
|  |  |  | 1 | -133 |
|  |  |  | 1 | -136 |
|  |  |  | 3 | WT |
| MSTN-sgRNA-2 on fibroblasts | - | positive | 5 | -1 |
|  |  |  | 1 | -11 |
|  |  |  | 1 | -26m1 |
|  |  |  | 1 | -158m3 |
|  |  |  | 8 | WT |
| FGF5-sgRNA-1 on fibroblasts | - | positive | 1 | -1 |
|  |  |  | 1 | +1 |
|  |  |  | 1 | +1 |
|  |  |  | 1 | -2 |
|  |  |  | 1 | -4 |
|  |  |  | 1 | -5 |
|  |  |  | 1 | -6 |
|  |  |  | 1 | -6 |
|  |  |  | 1 | -7 |
|  |  |  | 1 | -12 |
|  |  |  | 1 | -36+31 |
|  |  |  | 4 | WT |
| FGF5-sgRNA-2 on fibroblasts | - | positive | 1 | -1 |
|  |  |  | 2 | -2 |
|  |  |  | 1 | +2 |
|  |  |  | 1 | -3 |
|  |  |  | 1 | -6 |
|  |  |  | 1 | -8 |
|  |  |  | 1 | -7m1 |
|  |  |  | 1 | -27 |
|  |  |  | 1 | +32 |
|  |  |  | 1 | +54 |
|  |  |  | 1 | -198 |
|  |  |  | 4 | WT |
| FGF5-sgRNA-1 and 2 on fibroblasts | - | positive | 1 | -5 |
|  |  |  | 1 | -1, -6m2 |
|  |  |  | 1 | -13, -6 |
|  |  |  | 4 | -169 |
|  |  |  | 1 | t169 |
|  |  |  | 1 | m1t168 |
|  |  |  | 1 | -205+85 |
|  |  |  | 1 | -169+271 |
|  |  |  | 7 | WT |

**Supplementary Table S2** List of Putative Off-Target Sites Homologous to sgRNAs.


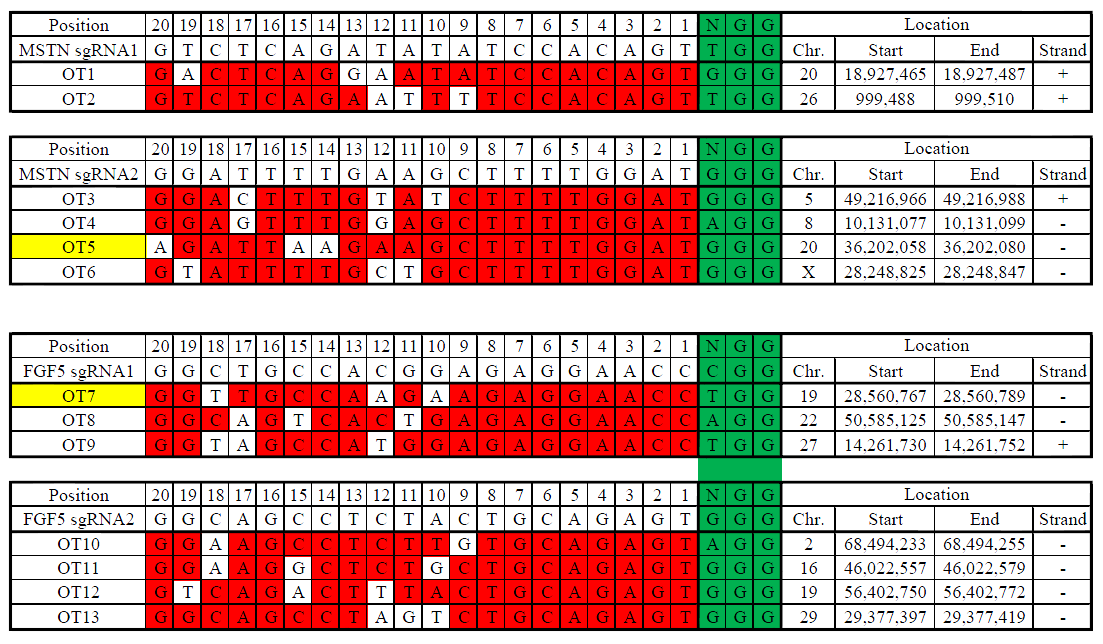


The PAM (NGG) sequences are marked in green. Based identical to sgRNAs are shaded in red. Confirmed off-target mutations are shaded in yellow.

**Supplementary Table S3** Sequencing and T7E1 analysis summary of off-target on fibroblasts.

| **No.** | **PCR product sequencing** | **T7E1** | **Sequenced Colonies** | **Mutations** |
| --- | --- | --- | --- | --- |
| MSTN-sgRNA-1 off-target site 1 on fibroblasts | SP | negative | - | WT |
| MSTN-sgRNA-1 off-target site 2 on fibroblasts | SP | negative | - | WT |
| MSTN-sgRNA-2 off-target site 1 on fibroblasts | SP | negative | - | WT |
| MSTN-sgRNA-2 off-target site 2 on fibroblasts | SP | negative | - | WT |
| MSTN-sgRNA-2 off-target site 3 on fibroblasts | SP | positive | 1 | -1 |
|  |  |  | 1 | -94 |
|  |  |  | 21 | WT |
| MSTN-sgRNA-2 off-target site 4 on fibroblasts | SP | negative | - | WT |
| FGF5-sgRNA-1 off-target site 1 on fibroblasts | MP | positive | 6 | -1 |
|  |  |  | 2 | -2 |
|  |  |  | 1 | -25 |
|  |  |  | 1 | -81 |
|  |  |  | 15 | WT |
| FGF5-sgRNA-1 off-target site 2 on fibroblasts | SP | negative | - | WT |
| FGF5-sgRNA-1 off-target site 3 on fibroblasts | SP | negative | - | WT |
| FGF5-sgRNA-2 off-target site 1 on fibroblasts | SP | negative | - | WT |
| FGF5-sgRNA-2 off-target site 2 on fibroblasts | SP | negative | - | WT |
| FGF5-sgRNA-2 off-target site 3 on fibroblasts | SP | negative | - | WT |
| FGF5-sgRNA-2 off-target site 4 on fibroblasts | SP | negative | - | WT |

**Supplementary Table S4** Oligonucleotides for generating sgRNA expression vectors

| MSTN sgRNA1 top strand | tagGTCTCAGATATATCCACAGT |
| --- | --- |
| MSTN sgRNA1 bottom strand | aaacACTGTGGATATATCTGAGA |
| MSTN sgRNA2 top strand | TAGGATTTTGAAGCTTTTGGAT |
| MSTN sgRNA2 bottom strand | aaacATCCAAAAGCTTCAAAAT |
| FGF5 sgRNA1 top strand | taGGCTGCCACGGAGAGGAACC |
| FGF5 sgRNA1 bottom strand | aaacGGTTCCTCTCCGTGGCAG |
| FGF5 sgRNA2 top strand | taGGCAGCCTCTACTGCAGAGT |
| FGF5 sgRNA2 bottom strand | aaacACTCTGCAGTAGAGGCTG |

**Supplementary Table S5** The information of sgRNAs in the goat *MSTN* and *FGF5* genes.

| **sgRNA** | **Targeting site** | **Location** | **Strand** |
| --- | --- | --- | --- |
| MSTN sgRNA-1 | GTCTCAGATATATCCACAGTTGG | Chr2:6316212-6316234 | - |
| MSTN sgRNA-2 | GGATTTTGAAGCTTTTGGATGGG | Chr2:6318642-6318664 | + |
| FGF5 sgRNA-1 | GGCTGCCACGGAGAGGAACCCGG | Chr6:92103586-92103608 | + |
| FGF5 sgRNA-2 | GGCAGCCTCTACTGCAGAGTGGG | Chr6:92103755-92103777 | + |

**Supplementary Table S6** Primers for genotyping and amplifying Cas9/sgRNA targeted *FGF5* and *MSTN* fragment.

| **Gene** | **Name of primer** | **Sequence** | **Amplicon (bp)** |
| --- | --- | --- | --- |
| MSTN | MSTN E2 Forward | GACATGGAGGCGTTCGTTCATT | 422 |
|  | MSTN E2 Reverse | CTGGGAAGGTTACAGCAAGATCA |  |
|  | MSTN E3 Forward | TAGAAGTCAAGGTAACAGACAC | 509 |
|  | MSTN E3 Reverse | GTTCATATACTGTAGCTTGTGC |  |
| FGF5 | FGF5 Forward | CCAACCCTGCAAGATGCACTTA | 528 |
|  | FGF5 Reverse | TTCTGGAGGAGAGCAAGCAACT |  |

**Supplementary Table S7** Sequences of primers for PCR amplification of off-target sites.

| **Primer pair** | **Putative Off-Target Site** | **Relative sgRNA** | **Primer sequence** | **Amplicon** |
| --- | --- | --- | --- | --- |
| PP1 | OT1 | MSTN-sgRNA1 | TGACCTTCTGGAGATACGAATC | 664 bp |
|  |  |  | CTAGTAACACTGACGTACACAAG |  |
| PP2 | OT2 |  | TCCTTCATGGTGCAAGCGTCTT | 602 bp |
|  |  |  | CCAACATCCACTGGCTCATAGAA |  |
| PP3 | OT3 | MSTN-sgRNA2 | GCTTATGCGGAAGTGTTGCCAGA | 711 bp |
|  |  |  | AGCAGAACCAACAGCCCTACTCA |  |
| PP4 | OT4 |  | CCAAGCAAGAAGACTGTAGTGAG | 737 bp |
|  |  |  | TCAATGGAATGACGACGACCTT |  |
| PP5 | OT5 |  | CTTAGAGGGCACAATCAGAACC | 706 bp* |
|  |  |  | CCATCACCAGTTACATTCACAGT |  |
| PP6 | OT6 |  | ATACATGCTGCTTACAGGAGTCT | 500 bp |
|  |  |  | CACCTATTCCAGTCCACCTTAGT |  |
| PP7 | OT7 | FGF5-sgRNA1 | TTCCATAGGCTGCGTTAGTCTA | 780 bp |
|  |  |  | GGAACTGTATTCAACATCCTGTG |  |
| PP8 | OT8 |  | GCTTTGAGTGATCCTTCTGGTT | 683 bp |
|  |  |  | CCTCACAAGAAGATGCAAGACAA |  |
| PP9 | OT9 |  | GCAGACAGCATTATGTTGATTGG | 638 bp |
|  |  |  | TGATTGTAACTCGCAGTCCTAAG |  |
| PP10 | OT10 | FGF5-sgRNA2 | TGGATCAGGTAGAGCAATGGAT | 575 bp |
|  |  |  | AAGGCTTAGTCTGCCATCTTCT |  |
| PP11 | OT11 |  | CCTTAGTACAGTTGAGCTGACAT | 713 bp |
|  |  |  | TCCAATGATACCGACTCCAGAT |  |
| PP12 | OT12 |  | TGTTTCTGAGGGAAGGCATCCT | 572 bp |
|  |  |  | CGAGAAGACACTGACCACTCCA |  |
| PP13 | OT13 |  | ACCAGTTCAAGAACATCTGCTT | 607 bp |
|  |  |  | CATCATAGACCACAAGACAGGAT |  |
